# Anaerobic Treatment of the Liquid Fraction of Food Waste in a Hybrid Reactor with Spatially Structured Biomass: Process Performance and Microbial Community Dynamics

**DOI:** 10.1021/acsomega.5c11752

**Published:** 2026-03-19

**Authors:** Adriana Alves Barbosa, Isabelli Dias Bassin, Camila Pesci Pereira, Douglas Alfradique Monteiro, Caio Tavora Coelho da Costa Rachid, João Paulo Bassin

**Affiliations:** † Chemical Engineering Program, COPPE, 28125 Federal University of Rio de Janeiro, Rio de Janeiro, RJ 21941-972, Brazil; ‡ Biochemical Engineering Department, School of Chemistry, Federal University of Rio de Janeiro, Rio de Janeiro, RJ 21941-972, Brazil; § Department of General Microbiology, Institute of Microbiology, Federal University of Rio de Janeiro, Rio de Janeiro 21941-972, Brazil; ∥ Civil Engineering Program, COPPE, Federal University of Rio de Janeiro, Rio de Janeiro, RJ 21941-972, Brazil

## Abstract

This study investigated the performance of a hybrid upflow anaerobic reactor (HUAR), integrating suspended and fixed biomass, for the treatment of the liquid fraction of food waste (LFFW), a nutrient-rich and highly organic strength stream generated during municipal organic waste handling. The reactor was fed with LFFW diluted to 10, 15, 30, 50, and 75% (v/v), corresponding to organic loading rates (OLRs) of 2.9, 4.1, 7.4, 11.5, and 19.2 kg COD/(m^3^·d), respectively, at a constant hydraulic retention time of 48 h. COD removal efficiency increased with OLRs, reaching 81% at the highest LFFW content, while methane content in biogas rose from 52 to 73%. Process stability was maintained throughout the reactor operation, with pH, alkalinity, and VFA/TA ratios remaining within optimal ranges (<0.4), and intermediate VFA accumulation was effectively buffered. Microbial community profiling revealed microbial functional stratification between suspended and attached biomass. Bacteria dominated the suspended phase (76%), driving hydrolysis and acidogenesis, while the biofilm zone had a higher proportion of methanogenic Archaea (35%), favoring methane production. The attached biomass was enriched in Methanothrix and Methanosarcina, along with syntrophic bacteria such as Macellibacteroides, Aminivibrio, and Cloacibacillus. The dual-compartment design promoted stable operation and efficient conversion of organics to methane even at high organic loading rates. These results demonstrate that the HUAR is a robust and high-performance system for anaerobic treatment of the liquid fraction of food waste, offering promising opportunities for integration into circular economy strategies and decentralized waste-to-energy solutions

## Graphical abstract

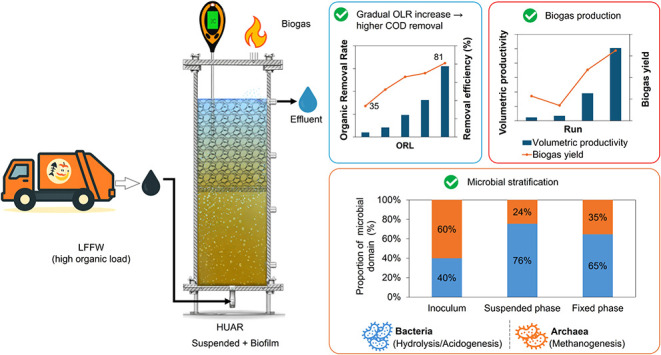

## 1 Introduction

In 2022, a report prepared by the World Bank, referring to global waste generation in 2016 with projections for 2030 and 2050, revealed that more than two million tons of waste were generated in 2016, with forecasts indicating a 70% increase by 2050. Brazil was identified as the largest producer of urban solid waste in Latin America and the Caribbean, with a generation of 81.8 million tons of solid waste in 2022, with projections indicating that the country will produce one hundred million tons by 2030.^1^ The gravimetric composition of solid urban waste generated in 2020 was 45.3% organic material, 16.8% plastic, 14.1% metals, 10.4% paper and cardboard, with the remaining proportion being other materials.

Globally and in Brazil, organic waste, primarily consisting of food scraps and gardening waste, accounts for nearly half of the municipal solid waste (MSW) generated.^1^ The organic fraction of municipal solid waste (OFMSW) is predominantly composed of food scraps, garden waste, paper, and cardboard, which constitute the organic solid fraction, in addition to the water fraction. The relative proportion between dry matter and moisture can vary significantly depending on factors such as seasonality, climate, population habits, and waste collection and storage practices.^2–4^

The liquid fraction of food waste (LFFW), which results from the compaction of food waste in organic waste collection trucks and is associated with the water content in OFMSW, has a high organic matter content, as well as nutrients like phosphorus and nitrogen. This fraction must be treated to minimize environmental impacts, such as eutrophication, groundwater contamination, and greenhouse gas emissions, while also enabling the potential recovery of valuable resources through appropriate treatment technologies.

One effective way to harness OFMSW for energy production and the generation of value-added products is through anaerobic digestion (AD). AD is a biological process that occurs in the absence of oxygen, where microorganisms break down organic matter through a series of biochemical reactions. This process produces a gaseous mixture primarily composed of methane and carbon dioxide, known as biogas, which has a high calorific value and can be utilized for energy generation. Additionally, the process results in the formation of digestate, which can exist in either solid or liquid form. Depending on its physical, chemical, and biological characteristics, digestate can be repurposed as a biofertilizer.^5^

AD has other benefits like low sludge production, no energy aeration, methane generation, and long biomass preservation, but faces challenges such as slow start-up, lower organic removal, odor formation, and sensitivity to low temperatures.^6^ Optimizing conditions is key, and AD stands out in the circular economy for reducing landfill waste and transport costs, with successful applications at different scales.^7,8^

Previous studies on anaerobic treatment of food waste through AD reported the use of various substrates, including fruit and vegetable fractions, and waste from cafeterias and restaurants.^9–12^ A range of reactor types and operating conditions have been explored, including batch and continuous systems and one- and two-stage configurations. Among the reactors investigated are the Upflow Anaerobic Sludge Blanket (UASB), Continuously Stirred Tank Reactor (CSTR), Anaerobic Sequencing Batch Reactor (ASBR), anaerobic filters (AF) and Internal Circulation (IC) reactors. However, studies specifically addressing the treatment of the LFFW through AD remain limited. Existing research has primarily explored its treatment for organic matter removal, as well as its potential for biogas production and volatile fatty acids recovery, using systems such as CSTR and UASB reactors.^13–16^

As the performance of anaerobic reactors relies strongly on efficient sludge retention and adequate contact between microbial biomass and the substrate,^17^ choosing an appropriate reactor configuration becomes a key design factor that significantly affects treatment efficiency, operational stability, and overall biogas production. Hybrid anaerobic reactors that combine suspended biomass and attached biofilm within a single system have gained increasing attention. Initially developed for treating high-strength industrial wastewater by combining UASB and AF concepts, hybrid systems integrate the advantages of suspended biomass, which favors biodegradation by enhancing microorganism-substrate contact, and immobilized (attached) biomass (biofilm), which increases solids retention time and reduces biomass loss at high hydraulic loads.^18–20^ Furthermore, hybrid anaerobic reactors can sustain multiple anaerobic metabolic pathways within a single system, enabling the coexistence of fermentative, acetogenic, and methanogenic processes, thereby enhancing operational robustness and supporting efficient organic matter removal and biogas production.^21^

Recent studies have demonstrated the high performance of hybrid anaerobic systems in both organic matter removal and bioenergy recovery. Serrano-Meza et al.^21^ showed that a single-stage upflow hybrid reactor promoted metabolic specialization between suspended and attached biomass during tequila vinasse treatment, enabling stable operation and the simultaneous production of hydrogen and methane. Microbial analyses revealed that fermentative bacteria dominated the suspended biomass, while methanogenic archaea were preferentially retained in the biofilm. Similarly, Chatterjee and Mazumder^22^ reported COD removal efficiencies above 90% in a three-stage upflow hybrid reactor treating fruit and vegetable waste at an OLR of ∼16 kg COD/(m^3^·d).

Despite these advances, the application of hybrid anaerobic reactors for treating the LFFW remains largely unexplored. This study, therefore, assessed the feasibility of LFFW treatment using an innovative hybrid reactor that combines suspended and attached biomass to enhance organic matter degradation, in contrast to previous research, which has primarily relied on conventional systems such as CSTR, UASB, and IC reactors for treating the solid fraction of OFMSW. In addition to assessing reactor performance, this work also assessed the microbial community dynamics, with a particular focus on methanogenic archaea, to provide mechanistic insights into methane production in LFFW treatment.

## 2 Materials and Methods

### 2.1 Feedstock

The LFFW used in this study, derived from the initial compaction of solid organic waste fractions by specialized compactor trucks used for selective collection, was collected every 15 days at the Waste Transfer Unit of the Municipal Urban Cleaning Company (COMLURB) in Rio de Janeiro, Brazil. The LFFW was evaluated through routine physicochemical analyses as pH, Chemical Oxygen Demand (COD), Biochemical Oxygen Demand (BOD), Total Suspended Solids (TSS), Volatile Suspended Solids (VSS), Volatile Fatty Acids (VFA), alkalinity, ammoniacal nitrogen and phosphorus. Collection occurred periodically, and the LFFW samples were stored under refrigeration at 1.8 °C to preserve their physical and chemical properties.

### 2.2 Anaerobic Reactor System

The experimental system consisted of an upflow hybrid anaerobic reactor (HUAR), with suspended and immobilized biomass, constructed from acrylic material. The reactor had a height of 34.0 cm and a diameter of 13.7 cm, with a total capacity of 5 L and a working volume of 4 L. The lower section of the reactor contained suspended biomass, while the upper section comprised adhered solids. The upper compartment was filled with 250 units of immobilization media, composed of polyurethane foam encased within a polypropylene structure (MiniBiobob, Bioproj, Brazil). This media offers a surface area higher than 1,200 m^2^/m^3^ for biofilm development.

The HUAR was operated in an acclimatized laboratory environment at a controlled temperature of 30 °C, with continuous feeding and a hydraulic retention time (HRT) of 48 h. The feed was introduced at the bottom of the reactor, in an ascending flow, with the aid of a peristaltic pump (Model BT100-2J, Longer Pump, China). The reactor was equipped with four side outlets to collect and analyze both suspended and attached biomass at various height levels. Additionally, a temperature and pH meter was installed at the top of the reactor, and hoses were connected to a gas meter (Ritter Milligascounter, Germany) for measuring the volume of biogas generated during the process, as illustrated in Figure 1.

**Figure 1:**
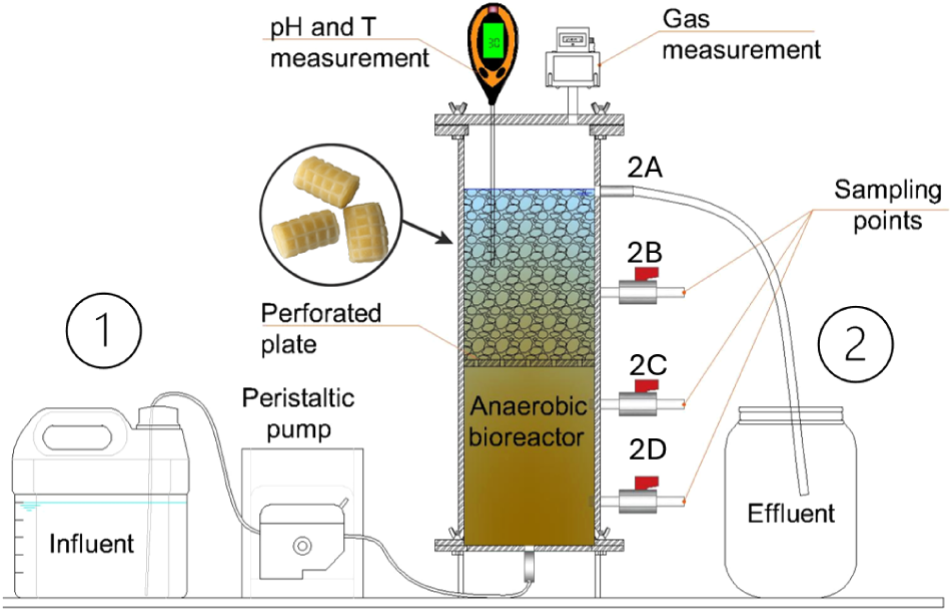
Schematic diagram of the experimental setup. (1) Container with liquid fraction from food waste (LFFW) diluted in tap water; (2) system components, including peristaltic pump; hybrid upflow anaerobic reactor (HUAR) containing suspended biomass (zones 2C and 2D) and immobilized biomass (zones 2A and 2B) and treated effluent container.

For the inoculation of the anaerobic reactor, a mixture of anaerobic sludge from brewery effluent treatment and excess sludge from a domestic wastewater treatment plant was used in a 3:1 ratio, respectively. The physicochemical characteristics of the inoculum were as follows: total solids concentration of 5.6 g/L, and volatile solids concentration of 4.4 g/L. The inoculum was introduced into the reactor at a volume corresponding to approximately 20% of the total reactor volume.

### 2.3 Experimental Procedures

The experiment was designed to evaluate the anaerobic treatment of LFFW, which, in its raw form, had a COD of around 60 g/L. The system feed initially consisted of 10% LFFW and 90% tap water, aiming to evaluate the contribution of organic matter exclusively from the substrate, without interference from other organic matrices. The proportion of LFFW in the feed stream was gradually raised to 75%, with a corresponding reduction in the water content. Under these conditions, the organic loading rate (OLR) was as follows: 2.93, 4.07, 7.38, 11.55, and 19.22 kgCOD/(m^3^·d) for experimental conditions 1, 2, 3, 4 and 5, which corresponds to 10, 15, 30, 50 and 75% of LFFW in the feeding stream, respectively. The feed pH was adjusted to a range of 6.5 to 7.5 using sodium bicarbonate. Each LFFW proportion was maintained for a sufficient period to achieve stable performance, defined as the point at which soluble COD (sCOD) removal and the VFA/TA ratio, a reliable indicator of anaerobic process stability, reached steady-state conditions. Once stability was reached, the LFFW proportion was increased. The duration of runs 1, 2, 3, 4, and 5 was 62, 83, 108, 157, and 311 days, respectively. The physicochemical characteristics of the raw LFFW and its diluted mixtures with water (at proportions of 10, 15, 30, 50, and 75%) are summarized in Table 1. The values presented in Table 1 represent average concentrations obtained from multiple influent samples collected throughout each experimental run. Influent characterization was conducted every 4–5 days in parallel with reactor monitoring, and the reported values correspond to the mean of all measurements collected over the respective run.

**Table 1:** Characteristics of Raw LFFW and its Diluting Forms Used for Reactor Feeding in Each Regime Evaluated

		Experimental runs (percentage of LFFW in the feeding)				
Parameter	LFFW (undiluted)	Run 1 (10%)	Run 2 (15%)	Run 3 (30%)	Run 4 (50%)	Run 5 (75%)
pH	4.1 ± 0.3	7.4 ± 0.3	7.1 ± 0.2	6.5 ± 0.3	6.4 ± 0.5	6.5 ± 0.5
VSS/TSS (%)	92	86	87	94	87	84
sCOD (g/L)	56.3 ± 3.8	5.9 ± 1.1	8.1 ± 0.6	14.8 ± 1.7	24.2 ± 3.2	38.4 ± 3.3
Ammonia (mgN/L)	438 ± 10	44 ± 23	82 ± 17	147 ± 30	286 ± 65	438 ± 136
Phosphorus (mgP/L)	540 ± 30	44 ± 9	37 ± 6	73 ± 31	361 ± 119	314 ± 64

### 2.4 Analytical Methods

The reactor performance was evaluated in terms of organic load removal, biogas production, and operational stability. During reactor operation, analyses of pH, total acidity, alkalinity, ammoniacal nitrogen (NH_4_^+^-N), Total Phosphorus (TP), chemical oxygen demand (COD), biological oxygen demand (BOD), total suspended solids (TSS), and volatile suspended solids (VSS) were frequently carried out. All analytical determinations were conducted following the procedures outlined in the Standard Methods for the Examination of Water and Wastewater.^23^ Detection of low molecular weight Volatile Fatty Acids (VFA) was obtained by a High-Performance Liquid Chromatography (HPLC), model 1200, by Agilent Technologies, using the Aminex HPX-87H column (300 × 7.8 mm with 9 μm particle size, manufactured by Bio-Rad). Identification of acids (formic, acetic, propionic, butyric, and valeric) was achieved by comparing their retention times to those of the respective standards. pH was monitored daily, while COD, ammoniacal nitrogen, and phosphorus were analyzed twice per week. TSS, VSS, VFA, and alkalinity were determined weekly. BOD was analyzed only at the time of sample collection at the treatment facility (COMLURB). All analyses were performed in triplicate to ensure the reliability of the results.

For the determination of suspended solids, liquid samples were regularly collected at both the influent and effluent of the reactor, and TSS and VSS contents were determined according to Standard Methods for the Examination of Water and Wastewater.^23^ The attached biomass was measured only at the end of the HUAR operation to prevent oxygen exposure, since sampling required opening the reactor to remove the representative samples of the carrier media. To determine total attached solids (TAS) and volatile attached solids (VAS), samples were taken from four points along the attached biomass compartment, beginning at the interface between the suspended and attached biomass zones (midsection of the reactor), continuing through the intermediate levels, and extending to the top outlet of the reactor. This procedure was performed to ensure the removal of a representative sample of the biofilm, as its concentration varies along the length of the support media layer. The biofilm was detached from the carriers by vigorous washing and mechanical agitation in distilled water, following procedures by Fonseca and Bassin.^24^

Biogas production was monitored using a gas meter (MilliGascounter, Ritter) and biogas quality was further assessed using a gas chromatograph, a Micro GC (model CP-4900, Varian), equipped with two parallel analytical channels, both with thermal conductivity detectors (TCD), with a 10 m Porapak Q (PPQ) column, identification and quantification of CH_4_, CO_2_, H_2_S.

### 2.5 Statistical Analysis

Statistical comparisons among operational runs were performed based on daily overall COD removal efficiency using one-way analysis of variance (ANOVA). When significant differences were detected, results were interpreted at a significance level of α = 0.05. Analyses were performed using Microsoft Excel 2021 (version 2110).

### 2.6 DNA Extraction, 16S rRNA Gene Sequencing, and Bioinformatic Analysis

To analyze the microbial community in the HUAR at 30% LFFW, DNA was extracted from approximately 250 mg of biomass collected at the end of Run 3 using the DNeasy PowerSoil Kit (QIAGEN, USA). Cell lysis was performed with two cycles in a FASTPREP-24 5G system (MP Biomedicals, USA) at 6.0 m/s for 40 s each. A negative control was included in the extraction. DNA quality was assessed through 0.8% agarose gel electrophoresis, and DNA concentration was measured with a Qubit 4 fluorometer (Thermo Fisher Scientific, USA). The extracted DNA was amplified by polymerase chain reaction (PCR), targeting the V3–V4 region of the 16S rRNA gene using primers recommended by Zymo Research (341F (CCTACGGGDGGCWGCAG/CCTAYGGGGYGCWGCAG) and 806R (GACTACNVGGGTMTCTAATCC)), generating an approximately 460 bp fragment. Library preparation was performed with the Quick-16STM Plus NGS Library Prep Kit (V3–V4, Zymo Research), and sequencing was conducted on an Illumina MiSeq platform.

For comparison, sequencing of the V4 region of the 16S rRNA gene was also performed by Novogene (www.novogene.com, USA) using primers 515F (GTGCCAGCMGCCGCGGTAA) and 806R (GGACTACHVGGGTWTCTAAT)^25^ on an Illumina NovaSeq 6000 platform, generating 100 K paired-end reads.

## 3 Results and Discussion

### 3.1 Organic Matter Removal at Increasing Organic Loading Rates

The organic matter concentration at the inlet and outlet of the HUAR, in terms of soluble COD (sCOD), for each experimental run, is presented in Figure 2. The raw LFFW had an average sCOD of 56 g/L, while the average sCOD values for the HUAR influent were 5.9 g/L for Run 1, 8.1 g/L for Run 2, 14.8 g/L for Run 3, 24.2 g/L for Run 4, and 38.4 g/L for Run 5. The average sCOD values at the reactor outlet were 3.8 ± 1.3 g/L, 3.9 ± 1.4 g/L, 5.1 ± 2.1 g/L, 6.8 ± 1.9 g/L, and 6.1 ± 1.9 g/L for runs 1, 2, 3, 4, and 5, respectively.

**Figure 2:**
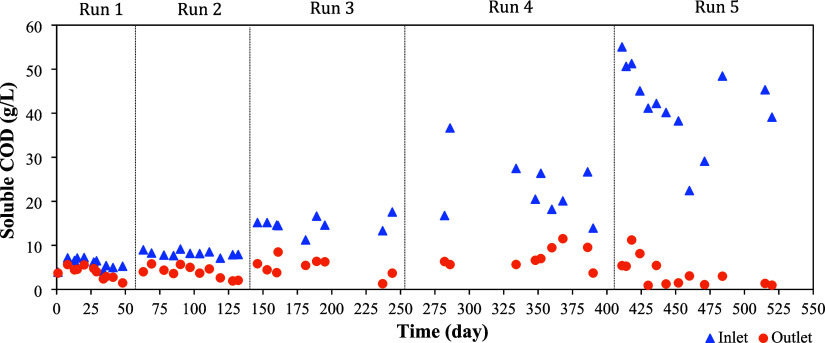
Soluble organic matter concentration at the inlet and outlet over the operation of HUAR from Run 1 (10% LFFW) to Run 5 (75% LFFW).

Based on the sCOD concentrations measured at the inlet and outlet of the HUAR, the average organic matter removal efficiency increased progressively across the operational runs: 35% in Run 1, 52% in Run 2, 66% in Run 3, 71% in Run 4, and reached 81% in Run 5. Overall COD removal efficiency differed significantly among runs (one-way ANOVA, *p* < 0.001), increasing progressively from Run 1 to Run 5. According to Chernicharo,^18^ anaerobic reactors typically remove between 40 and 70% of the influent organic material. Therefore, the HUAR has proven to be quite effective for organic matter removal at an HRT of 2 days (48 h). Another factor that may have contributed to the high organic matter removal was the reactor dual-compartment design, which combined suspended biomass in the lower section with attached biomass on support media in the upper section, enhancing both microbial activity and improving the interaction between the biomass and the organic substrate. According to previous studies,^21,26^ spatial separation of suspended and attached biomass in hybrid anaerobic reactors can be associated with metabolic specialization, thereby contributing to greater operational stability and higher biological activity.

To better understand the anaerobic organic matter degradation process along the reactor, samples were collected from different sampling points. Table 2 presents the sCOD values throughout the AD process in the HUAR for Runs 3, 4, and 5 (30%, 50%, and 75% LFFW, respectively).

**Table 2:** Average sCOD Values at Different Sampling Points in the Suspended and Attached Biomass Compartments of the HUAR for Runs 3 (30%), 4 (50%), and 5 (75%)

	Soluble COD (g/L)		
Samples	Run 3	Run 4	Run 5
Influent	14.7 ± 1.7	24.2 ± 3, 2	38.4 ± 4.5
2D	6.7 ± 1.2	10.9 ± 2, 9	11.9 ± 1.9
2C^a^	6.3 ± 1.2	9.8 ± 2, 6	9.9 ± 1.9
2B	5.9 ± 1.3	7.6 ± 2, 3	7.9 ± 2.0
2A^b^	5.0 ± 1.9	6.9 ± 2, 1	6.1 ± 1.1

a Sampling point at the outlet of the lower compartment (suspended biomass).

b Sampling point at the outlet of the upper compartment (attached biomass).

As shown in Figure 1, the HUAR is fed in an upward flow, starting from point 2D up to point 2A. Based on the COD balance throughout the HUAR, considering the influent, the outlet of the lower compartment (suspended biomass), and the outlet of the upper compartment (attached biomass), approximately 80% of the organic matter was removed by the suspended biomass in the lower compartment, with the remainder degraded in the upper compartment containing the attached biomass. These results indicate that the majority of biogas was produced in the lower region of the reactor, primarily by the suspended biomass. Biogas production will be discussed in more detail later.

A notable increase in sCOD removal was observed in the transition zone between points 2C and 2B, which corresponds to the interface between the suspended the immobilized biomass compartments. In this region, sCOD removal increased substantially from 5% in Run 3 to 22% in Run 4, maintaining a similar level of approximately 20% during the final experimental condition (Run 5).

The average COD removal efficiency was also evaluated as a function of the applied volumetric organic loading rate (OLR), accounting for influent sCOD and HRT. If the HUAR were fed with undiluted LFFW, the applied OLR would be 30 kgCOD/(m^3^·d). According to Lier et al.,^27^ anaerobic reactors can tolerate organic loads in the range of 5 to 35 kgCOD/(m^3^·d). Therefore, the use of diluted LFFW was preferred during the reactor startup to avoid organic overloading and operational instability. Figure 3 shows the relationship between the applied load and the removed load for each operational run of the HUAR. An increase in organic matter removal was observed as the OLR in the reactor increased, starting at 35% in Run 1 (10% LFFW) and reaching 81% in the last operating regime (75% LFFW). This result is possibly associated with the long acclimation process, with gradual percentage increases in the substrate. Similarly, Serrano-Meza et al.^21^ carried out an acclimation stage for the anaerobic treatment of tequila vinasse and observed a gradual increase in COD removal as the vinasse proportion, and consequently the OLR, was increased. Moreover, the incorporation of an upper compartment filled with support material enhanced biomass retention within the HUAR, thereby creating favorable conditions for the development of slow-growing microorganisms, such as methanogenic archaea, as discussed later.

**Figure 3:**
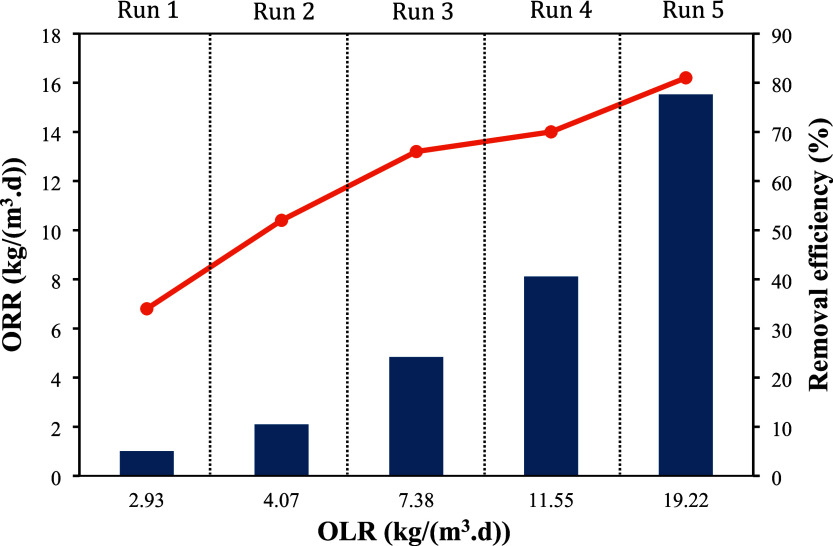
Organic loading rate (OLR) and organic removal rate (ORR) for experimental runs 1 to 5 (Bars—ORR; line—removal efficiency).

The overall characteristics of influent and effluent streams of the HUAR from Runs 1–5 in terms of physicochemical parameters are shown in Table S1 (Supporting Information). Complementary BOD_5_ analyses were also conducted during Run 4 (50% LFFW) to compare with COD results and, therefore, assess the biodegradable fraction of organic matter in both the influent and effluent of the HUAR (Table S2). The COD/BOD_5_ ratio was calculated as 1.8 for the influent and 3.2 for the treated effluent. According to Von Sperling,^28^ a COD/BOD_5_ ratio below 2.5 indicates a high proportion of biodegradable organic matter, suggesting the suitability of biological treatment processes. As noted by Kayhanian et al.,^29^ food waste typically contains a large fraction of easily degradable organic compounds. This was corroborated by the low COD/BOD_5_ ratio observed in the influent, confirming the high biodegradability of LFFW and supporting its potential for biological treatment.

### 3.2 pH, Acidity, and Alkalinity

The LFFW is naturally acidic, with an average pH of 4.04 (±0.30). Prior to buffering, the pH values of the diluted feed solutions were 4.19 ± 0.22, 3.92 ± 0.06, 3.97 ± 0.17, 4.15 ± 0.50, and 4.22 ± 0.09 for LFFW concentrations of 10, 15, 30, 50, and 75%, respectively. To ensure suitable conditions for anaerobic digestion, the feed stream was buffered to stabilize the pH and maintain process efficiency. Initially, a 6 M NaOH solution was used to adjust the feed pH to keep it within the range of 6.5 to 7.5. However, the pH did not remain stable and varied to values above 7.5. Therefore, powdered sodium bicarbonate (NaHCO_3_) was chosen for pH correction. With this compound, which has high solubility in water, it was possible to maintain the pH within the desired range (6.5 to 7.5). According to Valença et al.,^30^ many authors reported that sodium bicarbonate is more efficient for buffering anaerobic reactors, as it can easily neutralize excess protons, thereby preventing sharp pH drops caused by the accumulation of organic acids from food waste. This buffering action helps maintain conditions favorable for methanogenesis, supporting stable methane production.

The pH monitoring was carried out at four sampling points of the HUAR during the five operational runs. In general, pH values increased along the reactor (from bottom to top—points 2D to 2A); however, they remained within the optimal range for the AD, that is, between pH 6.0 and 8.0, which favors methane production.^31^ Alkalinity and acidity were also monitored in all regimes, and the results are shown in Table S3. The VFA/TA ratio is important to assess system stability. According to Chernicharo,^18^ the recommended value for VFA/TA ratio should be below 0.3. However, different values should be analyzed on a case-by-case basis, as they depend on the nature of the substrate and the characteristics of the reactor. As pointed out by Chen et al.,^32^ the range between 0.3 and 0.4 is considered optimal, but it is very important to assess whether this alkalinity-to-acidity ratio remains stable throughout the process. If it does not, corrective measures must be taken. Throughout the operation of the HUAR, the VFA/TA ratio remained consistently below 0.4, indicating effective buffering capacity and stable reactor performance despite increasing OLRs. Specifically, during runs 4 and 5, corresponding to 50% and 75% LFFW feed concentrations, this ratio stayed below 0.4 at all sampling points (2D to 2A). This stability can be largely attributed to the controlled addition of sodium bicarbonate, which effectively neutralized the inherent acidity of the LFFW substrate, thereby maintaining optimal pH conditions essential for efficient anaerobic digestion.

### 3.3 Assessment of Volatile Fatty Acids

The main low-molecular-weight fatty acids generated during the anaerobic treatment of LFFW were analyzed by HPLC in HUAR samples collected under operational runs with 30%, 50%, and 75% LFFW concentrations. The chromatographic profiles of the raw LFFW and samples from points 2C and 2D (suspended biomass) were similar, with notably higher acetic acid concentrations detected in the 30% and 50% LFFW (Runs 3 and 4). In contrast, during Run 5, samples from 2C and 2D showed the production of butyric and valeric acids. This finding was corroborated by chromatograms from samples 2A and 2B (fixed biomass). Besides displaying similar chromatographic profiles, they indicated the presence of additional acids such as acetic, propionic, and butyric acids.

These results suggest that, with the increased OLR throughout the experimental runs −5, there was no complete conversion of the intermediate VFAs to acetic acid. The accumulation of longer-chain VFAs, such as butyric and valeric acids, is often observed under higher OLR conditions, where the balance between acidogenesis and methanogenesis can be disrupted.^32,33^ In particular, methanogenic archaea may become inhibited or outpaced by acid-producing bacteria when substrate loading exceeds their metabolic capacity, leading to partial acid conversion and VFA buildup. Such accumulation can indicate a transient process imbalance but may also be stabilized with adequate buffering and microbial acclimation.^34^

### 3.4 Ammonia

Regarding ammoniacal nitrogen, raw LFFW presented an average concentration of 438 mg N/L. For the five operational runs (10, 15, 30, 50, and 75% LFFW), the average concentrations of ammoniacal nitrogen in the HUAR effluent samples were 122, 181, 252, 534, and 720 mg N-NH_4_^+^/L, respectively. During the AD process in the HUAR, hydrolysis of organic nitrogen may occur, breaking it down into simpler forms such as amino acids and peptides through the action of enzymes produced by microorganisms. These simple organic compounds can serve as substrates for organisms involved in the ammonification process, being converted into soluble ammonia, the inorganic form of nitrogen.^35^ Therefore, the ammonia concentration in the HUAR effluent corresponds to the sum of the ammonia already present in the influent (originating from the LFFW) and the ammonia produced through the ammonification of organic nitrogen. An increase in ammonia content was observed because of ammonification, and this increase becomes more pronounced throughout the operational runs, especially at run 5, with 75% LFFW, as shown in Figure 4.

**Figure 4:**
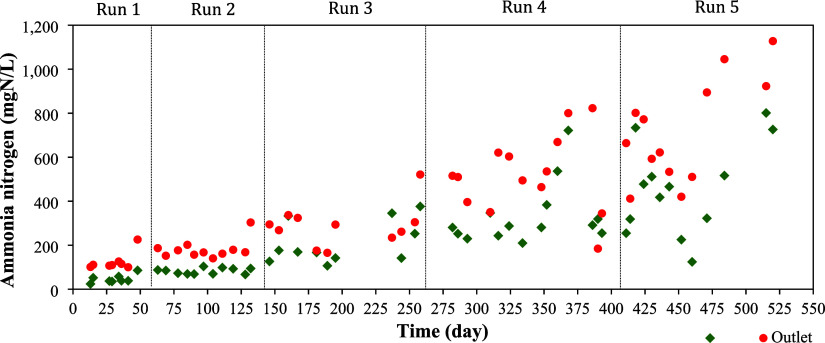
Ammonia at the inlet and outlet streams of HUAR for Runs 1–5.

### 3.5 Biogas Production and Composition

Figure 5 illustrates the effects of increasing the applied organic load on the system performance in terms of biogas production and yield throughout the different operational regimes.

**Figure 5:**
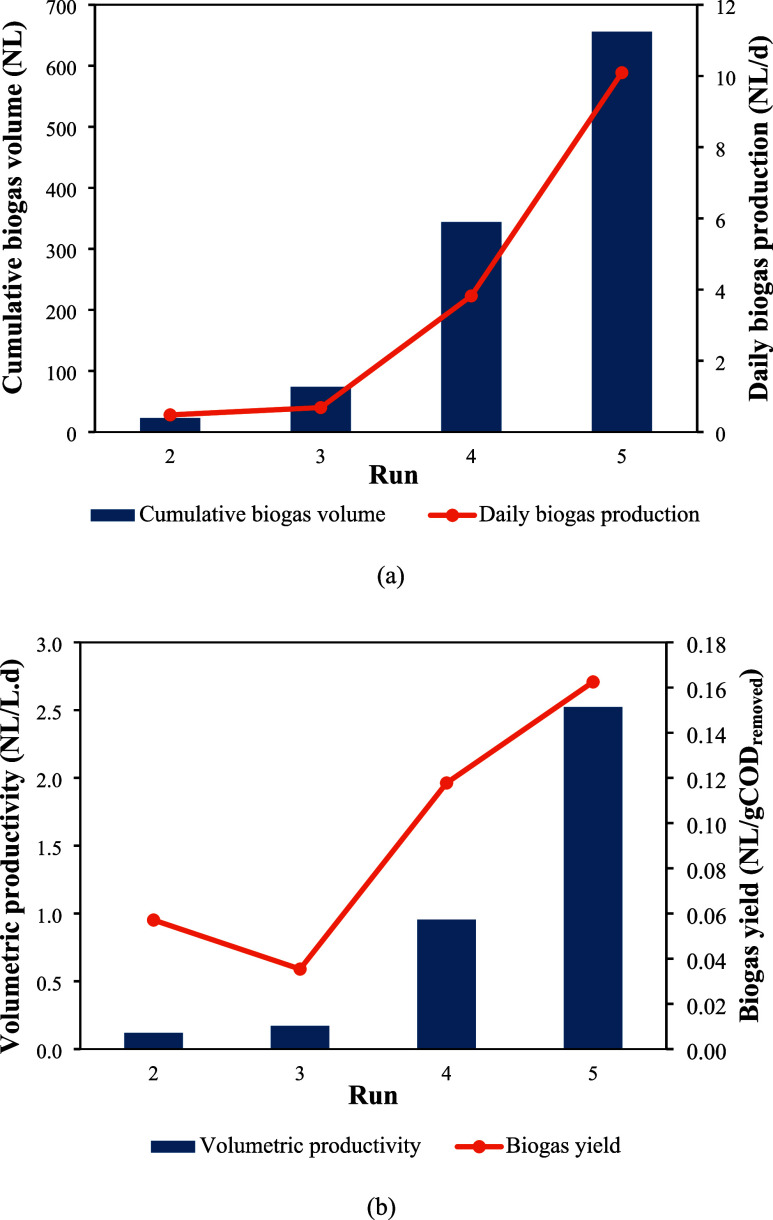
Biogas production (a) and yield (b) under varying organic loading rates across operational regimes.

A pronounced enhancement in both the cumulative biogas volume and the daily production rate was observed throughout the operational runs. Biogas output increased from approximately 25 NL and less than 1 NL/d in Run 2 to around 650 NL and over 10 NL/d in Run 5. Similarly, Meesap et al.^36^ reported daily biogas production between 2.1 and 6.4 NL/d in a hybrid reactor treating palm oil mill wastewater. This trend was accompanied by notable improvements in volumetric productivity and biogas yield, which rose from values below 0.12 NL/(L·d) and 0.06 NL/gCOD removed, respectively, to approximately 2.5 NL/(L·d) and 0.16 NL/gCOD removed in the final operational run. These results clearly indicate a substantial intensification of microbial activity, resulting in more effective conversion of the organic matter and improved utilization of the organic loading applied. Notably, the most marked performance gains from Run 4 onward suggest that the system achieved a phase of enhanced microbial adaptation. This improvement is likely associated with increased substrate availability and the establishment of more favorable environmental conditions for methanogenesis, contributing to greater process stability and overall operational efficiency.

However, despite the positive response of the system to the increase in organic load, the values obtained for specific biogas yield and volumetric productivity remained below those commonly reported in the literature for continuous anaerobic reactors operating with food waste. Several studies indicate productivities in the range of 3 to 6 NL/(L·d) and specific yields higher than 0.25 NL/g COD removed, mainly under optimized conditions of organic load, retention time and pH. For instance, Kim and Oh^37^ reported volumetric productivities of up to 5.0 NL/(L·d), while Qian et al.^38^ and other recent reviews confirm that specific yields above 0.25 NL/g COD removed are commonly achieved under stable mesophilic conditions and well-balanced substrate characteristics.

A low specific methane yield may be related to the intrinsic characteristics of anaerobic reactors and their operating conditions. In hybrid and compartmentalized systems, especially those treating high-strength wastewater, a portion of the removed COD can be diverted to nonmethanogenic pathways, including the formation of intermediates, biomass synthesis and maintenance. Additionally, methane can potentially remain dissolved in the liquid phase.^36,39–41^ Intanoo et al.^40^ evaluated a two-stage anaerobic reactor to enhance overall energy efficiency, aiming to maximize hydrogen production in the first stage, followed by methane conversion in the second stage. The authors reported a specific methane yield of 0.115 NL/g COD removed and a decrease in yield values as the organic load increased. Furthermore, directing organic matter toward the hydrogen production reduced the availability of substrate for subsequent methanogenesis. Serrano-Meza et al.^21^ evaluated a single-stage hybrid anaerobic reactor capable of simultaneously producing hydrogen and methane from tequila vinasse. In this system, metabolic separation favored fermentative pathways over methanogenesis, resulting in low specific methane yields (less than 0.01 NL CH_4_/COD removed). Similarly, in the present study, the combination of suspended and attached biomass in the hybrid reactor may favor metabolic specialization and process stability, but does not necessarily maximize the conversion of COD to methane, which explains the relatively low yield observed. It should also be noted that biofilm-based systems may promote greater methane retention due to mass-transfer limitations and microenvironment formation within the biofilm matrix, resulting in delayed methane release and increased dissolved methane fractions.^42^ Regarding the composition of the biogas produced in each run, the average methane content was 52.08% in Run 2, 62.8% in Run 3, 71.14% in Run 4, and 73.09% in Run 5. These values align with typical methane concentrations observed in anaerobic wastewater treatment systems, which generally range from 60% to 80%.^38^ In the study by Eusébio et al.,^43^ a hybrid anaerobic reactor exhibited robust biogas production even at elevated organic loads, achieving methane contents exceeding 77%.

### 3.6 Suspended and Attached Biomass Dynamics

The concentration of VSS at the reactor inlet was 628, 1,049, 1,619, 2,691, and 2,190 mg/L for the 10%, 15%, 30%, 50%, and 75% LFFW regimes, respectively. Corresponding effluent VSS concentrations were 397, 436, 457, 1,191, and 1,860 mg/L, indicating relatively stable retention of particulate organic matter across the operational phases. Notably, the VSS/TSS ratio consistently exceeded 70% in both influent and effluent streams throughout all loading conditions. This elevated VSS/TSS ratio highlights the predominance of organic over inert solids.

Regarding the solids within the HUAR, they consisted of immobilized biomass in the upper section, where support media were used to promote biofilm formation, and suspended biomass in the lower compartment. Both biomass fractions were influenced by the organic loading rate (OLR) applied to the reactor. The relatively high hydraulic retention time (HRT) of 2 days also played a key role in promoting the accumulation and retention of suspended sludge.^44,45^

To maintain the performance and stability of the anaerobic process, the support media in the HUAR were not removed during the operation, as doing so would have required opening the system, potentially introducing oxygen and disrupting the strictly anaerobic conditions. Therefore, the biofilm-containing media were only sampled at the end of the final run (75% LFFW). Consequently, the data presented refers exclusively to the attached solids collected during this phase. The average TAS was found to be 2.80 g/L, while the VAS corresponded to 1.41 g/L. Therefore, both the volatile and fixed solids contents were around 50%. The TAS and VAS expressed per area of carrier in Run 5 corresponded to 4.67 and 2.35 g/m^2^, respectively. Figure 6 illustrates the adhesion of solids to the HUAR support media.

**Figure 6:**
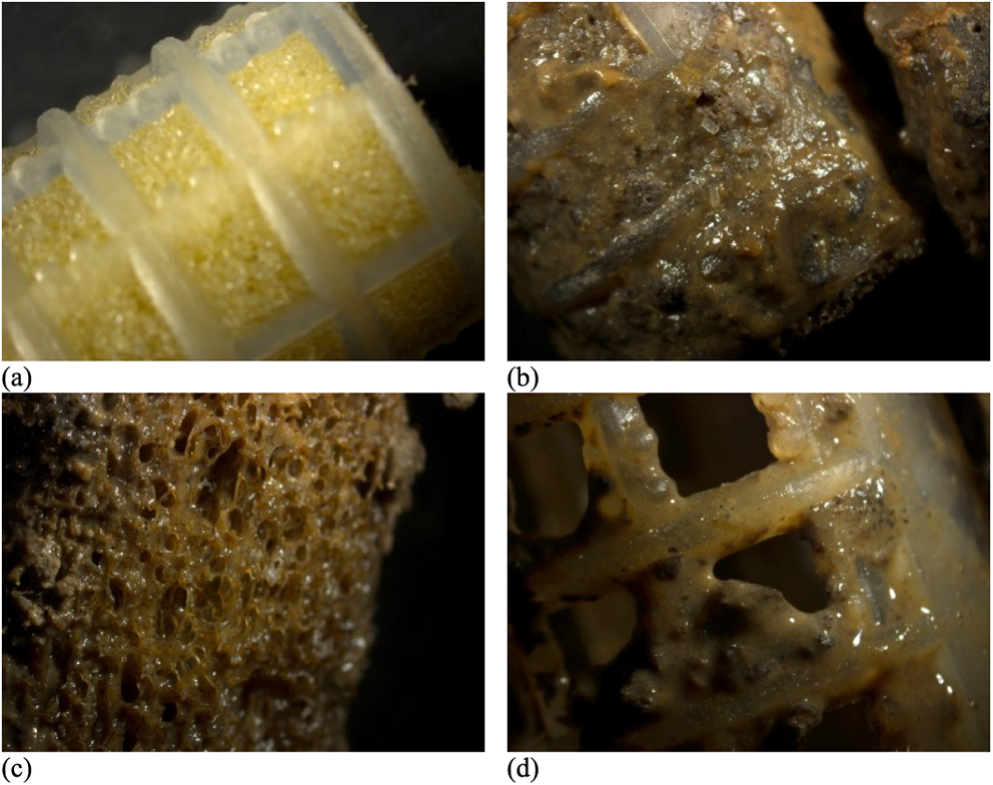
Stereomicroscope images of the media (BioBob) used in the HUAR. (a) BioBob before the beginning of reactor operation (without biofilm); (b) BioBob at the end of Run 5 (75% LFFW); (c) BioBob foam at the end of Run 5; (d) BioBob external plastic structure at the end of Run 5.

### 3.7 Microbial Community Assessment

To gain deeper insight into the anaerobic reactor operation, the microbial community was evaluated. Biomass samples were collected from both the suspended and attached biomass compartments of the HUAR during Run 3 (day 108) for comparative analysis. Additionally, inoculum biomass was also analyzed.

The taxonomic classification of the DNA sequences revealed only minor differences between the two compartments of the HUAR. Microbial richness and diversity were evaluated using the number of Operational Taxonomic Units (OTUs), the Chao richness estimator, and the Shannon and Simpson diversity indices, as summarized in Table 3.^46^ Similar richness profiles were observed in both reactor compartments, with 912 OTUs in the lower compartment and 957 OTUs in the upper compartment, indicating the role of the support media in immobilizing additional microbial taxa and resulting in an approximately 5% increase in OTU number. Consistently, the Chao index was higher in the compartment containing the support media, further confirming enhanced microbial richness in the attached-growth zone.

**Table 3:** Alpha Diversity Indices of Microbial Communities in the Inoculum, Suspended, and Attached Biomass in the HUAR

	Richness indexes		Diversity indexes	
Sample	Number of OTU	Chao	Shannon	Simpson
Inoculum	376	402	2.855	0.1959
Suspended phase	912	1,117	4.679	0.0291
Fixed phase	957	1,156	4.659	0.0352

Regarding diversity indices, lower Simpson values and higher Shannon values are indicative of greater microbial diversity. The Shannon index increases with both microbial richness and evenness, reflecting a more complex and diversified microbial community, while the Simpson index decreases as dominance declines.^46^ The similar Shannon and Simpson values observed in the two reactor compartments suggest comparable diversity levels, despite the slightly higher richness in the attached biomass. In contrast, comparison between the inoculum and the HUAR compartments shows that reactor operating conditions and substrate characteristics promoted a substantial increase in microbial richness and diversity, as evidenced by the markedly lower diversity indices observed in the inoculum.

Regarding the relative abundance of microbial domains, Bacteria and Archaea, the community composition in the inoculum consisted of approximately 60% Archaea and 40% Bacteria (Figure 7). This reflects the origin of the sludge from mature anaerobic digesters, where methanogenic Archaea typically dominate due to their crucial role in methane production. However, a significant shift was observed during reactor operation. In the suspended biomass compartment, Bacteria dominated the community, accounting for 76%, while Archaea decreased to 24%. This bacterial predominance aligns with their primary role in the initial stages of anaerobic digestion, including hydrolysis, acidogenesis, and acetogenesis, which involve the breakdown of complex substrates into volatile fatty acids and simpler compounds. Meesap et al.^36^ observed that bacteria predominated in suspended biomass in hybrid anaerobic reactors. The authors observed that, as the applied organic load increased gradually, intense hydrolysis and fermentation activity occurred in the sludge zone of the reactor, characterized by the predominance of bacteria.

**Figure 7:**
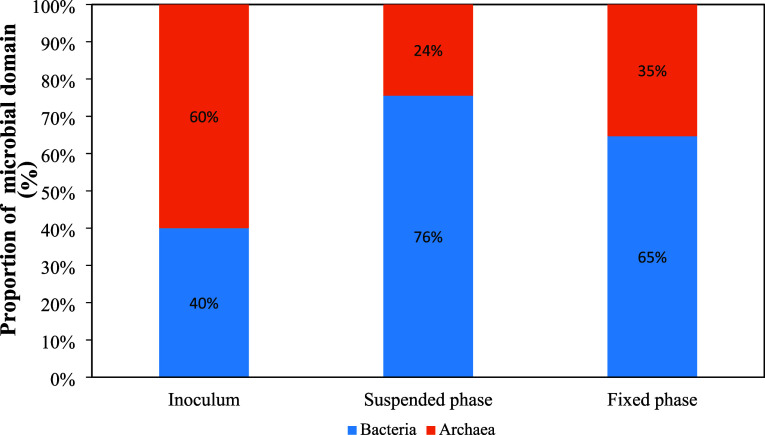
Relative abundance of Bacteria and Archaea in the inoculum as well as in the suspended and fixed biomass compartment within the HUAR.

In contrast, the upper zone of the HUAR, comprising biomass immobilized on support media, showed a more balanced distribution: 65% Bacteria and 35% Archaea (Figure 7). The higher proportion of Archaea in the fixed phase indicates that the support media favored the establishment and retention of slow-growing methanogens.^47,48^ Meesap et al.^36^ reported a high abundance of archaea within the microbial biofilm in the attached phase of the hybrid reactor. Both acetoclastic and hydrogenotrophic methanogenesis were observed in this biofilm, with the highest population density and activity in the fixed-bed zone. These findings suggest that, in hybrid anaerobic reactors, the suspended sludge and fixed-bed zones generally function as acidification and methanogenesis compartments, respectively. The structured biofilm environment likely offered protection against washout and environmental fluctuations, thereby creating a more favorable niche for these microorganisms. This compartmentalization suggests functional specialization within the reactor, where the suspended phase primarily facilitated rapid substrate breakdown, and the fixed phase promoted efficient methanogenesis. These findings support the importance of reactor design in enhancing microbial stratification and overall process stability in high-rate anaerobic systems.

The dominant phylum within the Archaea domain was Euryarchaeota (24% in the suspended phase and 35% in the media-attached phase), which includes a diverse group of thermophilic, aerobic, anaerobic, methanogenic, and halophilic organisms distinguished by their rRNA. Methanogenic archaea were responsible for methane production during the final stage of AD and are capable of consuming acetate and/or carbon dioxide and hydrogen, formate, alcohols, and methylated C1 compounds. Within the methanogenic Archaea domain, six phylogenetic orders were identified: *Methanosarcinales*, *Methanobacteriales*, *Methanomicrobiales*, *Methanococcales*, *Methanopyrales*, and *Methanocellales*.^49^ In HUAR, the most abundant orders in the media-attached compartment were *Methanosarcinales* (28%), *Methanobacteriales* (1%), and *Methanomicrobiales* (3%). Although these archaeal orders were already present in the inoculum introduced into the HUAR, an increase of 8%, 1%, and 2%, respectively, was observed in the immobilized biomass. Chatterjee and Mazumder^22^ observed Euryarchaeota as one of the three predominant phyla in methanogenic cultures in both the suspended and attached phases (21.8% and 23.9%, respectively) within a hybrid anaerobic digester.

A bacterial order that was absent in the inoculum and emerged in the HUAR, particularly in the suspended phase, was Lactobacillales, representing 6% of the microbial community. This order consists of Gram-positive bacteria capable of fermenting sugars into lactic acid.^50^ Desulfovibrionales (2% frequency) were also found in the attached biomass phase of the HUAR. These sulfate-reducing anaerobic bacteria oxidize organic compounds or molecular hydrogen, reducing sulfate to hydrogen sulfide,^51^ and may compete with methanogenic archaea for organic substrates. Serrano-Meza et al.^21^ detected sulfate-reducing bacteria (genus *Desulfovibrio*) in the attached biomass of the tequila vinasse treatment reactor at a frequency of approximately 2%. Fang et al.^52^ noted that a proportion below 3% of these organisms does not adversely affect the anaerobic digestion process. In contrast, Eusébio et al.^43^ and Meesap et al.^36^ did not report the presence of sulfate-reducing bacteria in their studies.

Three bacterial orders stood out in the HUAR: Clostridiales, Synergistales, and Bacteroidales. The second most abundant phylum in the HUAR was Firmicutes, which includes the class *Clostridia*—strictly anaerobic bacteria found at relative abundances of 13% and 14% in the suspended and attached biomass, respectively—confirming the maintenance of anaerobic conditions in the reactor.^53^ The third most abundant phylum was Synergistetes, comprising the class *Synergistia*. These Gram-negative, anaerobic bacteria were found at frequencies of 13% in the suspended phase and 14% in the attached phase of the HUAR. They have previously been reported in sludge and wastewater from anaerobic digesters, natural sources, seawater, sulfur mats, water from oil and gas production facilities, and host-associated microbiota.^54^

Sharing the same position (third), the phylum Bacteroidetes accounted for 14% of the microbial community in the attached phase of the HUAR. This phylum consists of Gram-negative, anaerobic bacteria that play a primary role in degrading biomass rich in complex carbohydrates. They are commonly found in soils and in the intestines of humans and animals, where processes like anaerobic digestion occur.^55^ Once again, the presence of this phylum confirms that operational conditions to maintain an anaerobic system were preserved. Figure 8 shows the relative abundance of phyla found in the inoculum and HUAR samples (suspended and attached biomass fractions).

**Figure 8:**
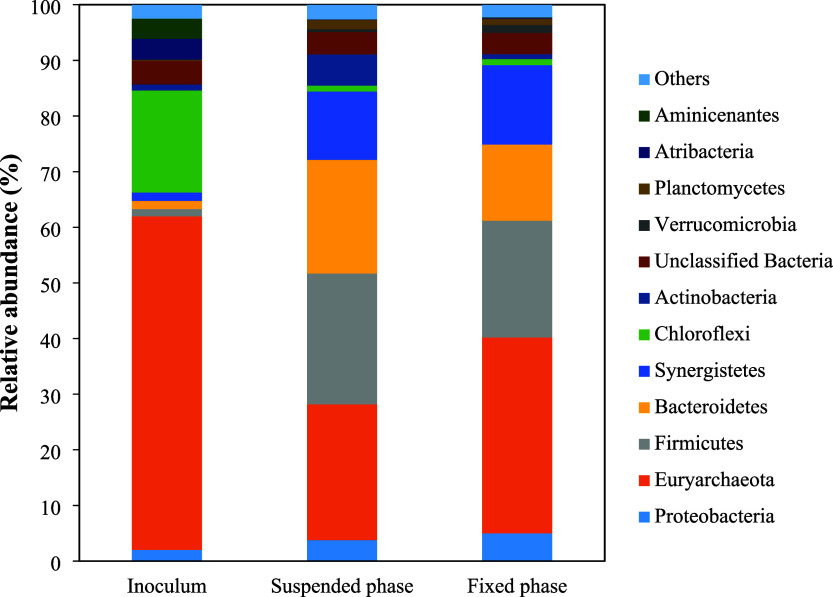
Relative abundance of phyla in the inoculum, as well as in the suspended and fixed biomass compartments of the HUAR.

The main microbial classes found in the samples analyses were Methanomicrobia, Anaerolineae, Methanobacteria, Deltaproteobacteria, Synergistia, Bacteroidia, and Clostridia (Figure 9). All these classes are representative of anaerobic organisms. Deltaproteobacteria may include both aerobic and strict anaerobic genera, such as the genus *Desulfovibrio*,^56^ which was found in the attached biomass zone of the HUAR.

**Figure 9:**
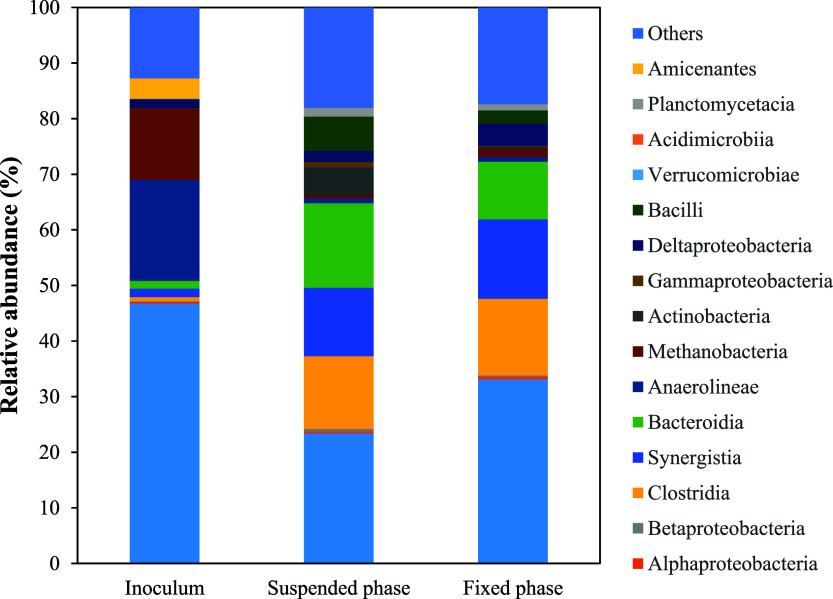
Relative abundance of class level in the inoculum, as well as in the suspended and fixed biomass compartments of the HUAR.

Methanomicrobia, a class of hydrogenotrophic archaea, contributes to methane production and exhibits the capacity for mixotrophic growth, oxidizing compounds ranging from cyclopentane and secondary alcohols to ethanol. Methane production generally occurs in biofilms, where direct interspecies hydrogen transfer enables efficient substrate conversion by the methanogenic microorganisms.^49^ In the HUAR, this class was found in greater abundance in the media-attached phase (relative abundance of 33%) compared to 23% in the suspended phase.

In the HUAR system, the dominant methanogenic family was Methanotrichaceae, represented solely by the genus *Methanothrix*.^57^ This acetoclastic archaea, which utilizes acetate as its exclusive energy source and cannot reduce carbon dioxide with hydrogen, was found in greater quantities in the inoculum and the attached solids zone of the anaerobic reactor, accounting for 43% and 14% of the microbial community, respectively (Figure 10). The second most abundant methanogenic genus was *Methanosarcina* (9%), known for its metabolic versatility, as it can produce methane via acetoclastic, hydrogenotrophic, and methylotrophic pathways. The genus *Methanosarcina* was found only in the reactor samples (not in the inoculum), corresponding to 9% of the microbial community. These organisms can utilize a variety of simple carbon substrates and perform methanogenesis via multiple pathways, including acetoclastic, carboxydotrophic, hydrogenotrophic, methylotrophic, and other methylated compound-based routes.^58^ Serrano-Meza et al.^21^ reported changes in the population dynamics of archaea, from the acclimation phase (high abundance of *Methanobacterium*) to the final analysis, with a marked increase in *Methanosarcina* during anaerobic treatment of vinasse. The authors claimed that *Methanosarcina* adapts better than other methanogenic archaea because it can produce methane via three methanogenic pathways.

**Figure 10:**
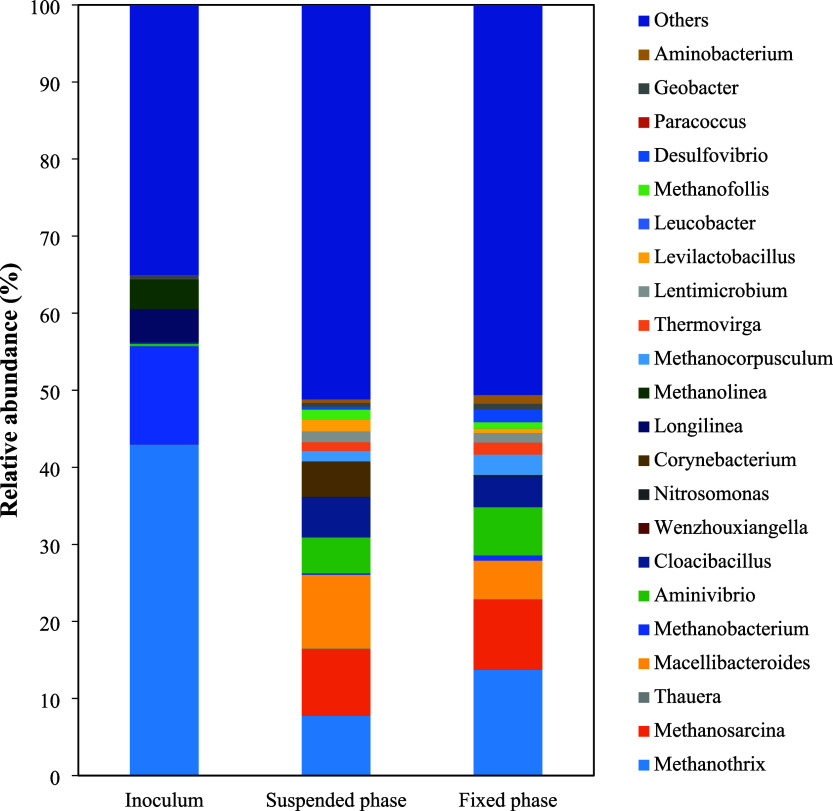
Genus-level microbial composition in the inoculum, suspended biomass, and attached biofilm fractions of the HUAR system.

Additional methanogenic archaea detected across various reactor compartments were *Methanobacterium*, *Methanolinea*, *Methanocorpusculum*, and *Methanofollis*. *Methanolinea* was found exclusively in the inoculum and is known to grow optimally at 20–40 °C, pH 6.5–7.4, using acetate.^59^ *Methanocorpusculum*, identified in the suspended biomass compartment, is a hydrogenotrophic methanogen,^60^ while *Methanofollis*, also present in the reactor, can utilize H_2_/CO_2_, formate, 2-propanol/CO_2_, and 2-butanol/CO_2_ under neutral pH and mesophilic conditions.^61^ The presence of *Methanobacterium* suggests potential hydrogenotrophic pathways.^36^

Besides the key genera associated with methanogenic pathways, *Methanothrix* and *Methanosarcina*, strictly anaerobic bacterial genera such as *Macellibacteroides*, *Aminivibrio*, and *Cloacibacillus* were also exclusively detected in the HUAR. These organisms are commonly involved in syntrophic relationships within AD processes and can metabolize organic acids and amino acids, suggesting favorable operational conditions within the reactor.^62,63^ Corynebacterium-like organisms, identified only in the adhered phase, represent facultative anaerobes capable of producing various organic acids from sugars.^64^ In addition, *Macellibacteroides*, belonging to the Bacteroidetes phylum and found predominantly in the adhered phase, are involved in carbohydrate degradation and the production of low molecular weight compounds.^65^

## 4 Conclusions

This study demonstrated the effectiveness and operational stability of a hybrid upflow anaerobic reactor (HUAR) for treating the liquid fraction of food waste (LFFW) under increasing organic loading rates (OLRs) up to around 20 kg COD/(m^3^·d). High COD removal efficiencies, up to 81%, were achieved even at elevated LFFW concentrations, confirming the reactor robustness and adaptability. Microbial community analysis highlighted the significance of the reactor hybrid design. The upper biofilm zone contained a 46% fraction of methanogenic Archaea, including hydrogenotrophic groups, supporting functional specialization, enhanced methane production, and improved process resilience. Key parameters, such as pH, VFA/alkalinity ratio, and gas composition, remained within optimal ranges. The HUAR system thus emerges as a robust, scalable solution for decentralized, energy-positive treatment of organic waste streams, aligning with circular economy principles and sustainable waste management goals.

## Supplementary Material



## Notes

Tables presenting the characteristics of the influent and effluent streams; COD and BOD_5_ values and their ratio; total alkalinity (g CaCO_3_/L); volatile fatty acids (g HAc/L); VFA/TA ratio at the four sampling points (PDF)
