# Supplementary material for: Anaerobic Treatment of the Liquid Fraction of Food Waste in a Hybrid Reactor with Spatially Structured Biomass: Process Performance and Microbial Community Dynamics
Source: ACS Omega. 2026 Mar 19;11(12):19111–23. doi: 10.1021/acsomega.5c11752 (PMC13044626; doi:10.1021/acsomega.5c11752)
Supplement: Supplementary file 1 [file ao5c11752_si_001.pdf]

Supplementary Material for: Anaerobic treatment of the liquid fraction of food waste in a hybrid reactor with spatially structured biomass: process performance and microbial community dynamics

Adriana Alves Barbosa[a], Isabelli Dias Bassin[b], Camila Pesci Pereira[a], Douglas Alfradique Monteiro[c], Caio Tavora Coelho da Costa Rachid[c], João Paulo Bassin[a,d*]

[a]Chemical Engineering Program, COPPE, Federal University of Rio de Janeiro, Rio de Janeiro, RJ, Brazil.

[b]Biochemical Engineering Department, School of Chemistry, Federal University of Rio de Janeiro, Rio de Janeiro, RJ, Brazil.

[c]Department of General Microbiology, Institute of Microbiology, Federal University of Rio de Janeiro, Rio de Janeiro, Brazil.

[d]Civil Engineering Program, COPPE, Federal University of Rio de Janeiro, Rio de Janeiro, RJ, Brazil.

*Corresponding author. Mailing address: Chemical Engineering Program/COPPE, Federal University of Rio de Janeiro, P.O. Box 68502, 21941-972, Rio de Janeiro, Brazil. Tel. +55 21 25628347, Fax +55 21 25628300, Email address: jbassin@peq.coppe.ufrj.br

S.1 Supplementary Tables

Table S1: Characteristics of influent and effluent streams of the HUAR from Runs 1 – 5.

**Parameters**	**Run 1 (10% LFFW)**		**Run 2 (15% LFFW)**		**Run 3 (30% LFFW)**		**Run 4 (50% LFFW)**		**Run 5 (75% LFFW)**	
	inlet	outlet	inlet	outlet	inlet	outlet	inlet	outlet	inlet	outlet
pH	7.4 ±0.3	7.8 ±0.4	7.1 ±0.2	7.8 ±0.1	6.5 ±0.3	8.1 ±0.3	6.4 ±0.5	7.9 ±0.4)	6.5 ±0.5	7.9 ±0.35
TSS (g/L)	0.73 ±0.08	0.56 ±0.02	1.21 ±0.05	0.53 ±0.02	1.72±0.13	0.57 ±0.04	3.10 ±0.32	1.40 ±0.06	3.68 ±0.33	2.50 ±0.10
VSS (g/L)	0.63 ±0.07	0.40 ±0.08	1.05 ±0.04	0.44 ±0.02	1.62 ±0.12	0.46 ±0.03	2.69 ±0.03	1.20 ±0.04	3.09 ±0.03	1.80 ±0.09
sCOD (g/L)	5.9 ±1.1	3.8 ±1.3	8.1 ±0.6	3.9 ±1.2	14.8 ±1.7	5.1 ±1.0	24.2 ±3.2	6.8 ±2.1	42.2 ±3.3	6.1±2.8
N- Ammoniacal (mgN/L)	44 ±23	121±49	82 ±17	181 ±53	147 ±30	252 ±69	286 ±65	534 ±186	438 ±136	926 ±173
Phosphorus (mg/L)	44 ±9	37 ±6	42 ±5	47 ±5	73 ±31	38 ±19	361 ±119	193 ±61	314 ±64	110 ±26
Total Alkalinity (g $CaCO_3$/L)*	4.3 ±0.09	5.1 ±0.09	5.4 ±0.05	5.4 ±0.06	6.4 ±0.06	8.0 ±0.06	7.9 ±0.09	10.9 ±0.3	7.8 ±0.06	10.8 ±0.1
Volatile Fatty Acids (g HAc/L)	1.2 ±0.02	1.5 ±0.02	1.8 ±0.05	2.2 ±0.03	3.8 ±0.03	3.4 ±0.04	3.4 ±0.04	4.1 ±0.1	3.6 ±0.03	4.2 ±0.03
VFA/TA	0.28	0.29	0.33	0.4	0.59	0.43	0.43	0.38	0.46	0.38

*Bicarbonate added

Table S2: COD and BOD_5 values and their ratio for run 4 (50% LFFW).

**Run 4**	**COD (g/L)**	**BOD_5 (g/L)**	**COD/BOD ratio**
inlet	35.82 ±2.96	19.55 ±0.94	1.8
outlet	4.62 ±1.64	1.42 ±0.50	3.2

Table S3: Total Alkalinity (g $CaCO_3$/L), VFA (g HAc/L) and VFA/TA ratio at the four sampling points of the HUAR ins Runs 1 – 5.

	**Run 1**			**Run 2**			**Run 3**			**Run 4**			**Run 5**		
	Alkalinity	VFA	VFA/TA	Alkalinity	VFA	VFA/TA	Alkalinity	VFA	VFA/TA	Alkalinity	VFA	VFA/TA	Alkalinity	VFA	VFA/TA
2A	5.14	1.55	0.30	5.44	1.84	0.34	8.05	2.93	0.36	10.91	4.14	0.38	10.85	4.25	0.39
2B	5.14	1.49	0.29	5.42	1.82	0.34	8.01	2.95	0.37	10.91	4.14	0.38	10.85	4.17	0.38
2C	5.13	1.38	0.27	5.37	1.88	0.35	7.97	2.94	0.37	10.83	4.12	0.38	10.78	4.14	0.38
2D	5.12	1.37	0.27	5.17	1.85	0.36	7.92	2.94	0.37	10.80	4.12	0.38	10.74	4.14	0.39
